# What funders are doing to assess the impact of their investments in health and biomedical research

**DOI:** 10.1186/s12961-022-00888-1

**Published:** 2022-08-09

**Authors:** Rachel Abudu, Kathryn Oliver, Annette Boaz

**Affiliations:** 1 Department of Public Health, Environments and Society, Faculty of Public Health Policy, London School of Hygiene and Tropical Medicine, London, United Kingdom; 2 Department of Health Services Research and Policy, Faculty of Public Health Policy, London School of Hygiene and Tropical Medicine, London, United Kingdom

**Keywords:** Funders, Research impact, Impact evaluation, Impact assessment methods, Impact assessment frameworks

## Abstract

As pressures to maximize research funding grow, biomedical research funders are increasingly tasked with demonstrating the long-term and real-world impacts of their funded research investments. Over the past three decades, research impact assessments (RIA) have emerged as an important tool for analysing the impacts of research by incorporating logic models, frameworks and indicators to track measures of knowledge production, capacity-building, development of research products, adoption of research into clinical guidelines and policies, and the realization of health, economic and social benefits. While there are currently several models for RIA within the literature, less attention has been paid to how funders can practically select and implement a RIA model to demonstrate the impacts of their own research portfolios. In this paper, a literature review was performed to understand (1) which research funders have performed RIAs of their research portfolios to date; (2) how funders have designed their assessments, including the models and tools they have used; (3) what challenges to and facilitators of success have funders found when adopting the RIA model to their own portfolio; and (4) who participates in the assessments. Forty-four papers from both published and grey literature were found to meet the review criteria and were examined in detail. There is a growing culture of RIA among funders, and included papers spanned a diverse set of funders from 10 countries or regions. Over half of funders (59.1%) used a framework to conduct their assessment, and a variety of methods for collecting impact data were reported. Issues of methodological rigour were observed across studies in the review, and this was related to numerous challenges funders faced in designing timely RIAs with quality impact data. Over a third of articles (36.4%) included input from stakeholders, yet only one article reported surveying patients and members of the public as part of the assessment. To advance RIA among funders, we offer several recommendations for increasing the methodological rigour of RIAs and suggestions for future research, and call for a careful reflection of the voices needed in an impact assessment to ensure that RIAs are having a meaningful impact on patients and the public.

**Supplementary Information:**

The online version contains supplementary material available at 10.1186/s12961-022-00888-1.

## Background

Amidst a background of competitive [1–3] and often political [4, 5] pressures to maximize research funding, biomedical research funders are increasingly asked to demonstrate the longer-term and real-world (health, economic and social) impacts of their research [6–9]. Consumers are eager to see value for money spent [10–12] and research gains translated into actionable and fully adopted policies that are evidence-informed and responsive to the needs of patients and the wider public [13, 14]. Over the last 30 years, research impact assessments (RIAs) have emerged as a leading tool for analysing the impacts of research by incorporating logic models, frameworks and indicators to track measures of knowledge production, capacity-building, development of research products, adoption of research into clinical guidelines and public policies, and the realization of health, economic and social benefits [8, 10, 15, 16].

Typically, RIAs enable the identification and assessment of research outputs, outcomes and impacts. These can be broadly understood to correlate with more traditional definitions of primary research outputs seen elsewhere in the literature. *Research outputs* are usually defined as citations/publications, and might be evidenced through case studies/research accomplishments, collaborations/networks; capacity-building/career advancement, future funding (applied or secured) or research targeting; and media citations/presentations. Secondary research outputs, also known as *outcomes*, might include products/research tools/patents/drugs and clinical practice/policy/commission memberships; and *impacts* include broader health, economic or societal downstream impacts of research [17].

While the availability of diverse models for RIA have been discussed at length in other reviews [9, 10, 18–21], less attention has been paid to the practical implementation of such RIA frameworks and models by research funders. Today, research funders have more RIA models at their disposal than ever [22], including but not limited to the Payback Framework [23], Canadian Academy of Health Sciences (CAHS) Framework [24], Canadian Health Services and Policy Research Alliance (CHSPRA) Informing Decision-Making Impact Framework [25], Research Impact Framework [26], Framework to Assess the Impact from Translational Health Research (FAIT) [27], Stryer’s four types of impact model [28], also known as the AHRQ impact model, Weiss’s Framework [29], Becker Medical Library Model [30], Social Impact Assessment Methods for Productive Interactions (SIAMPI) [31, 32] and Contribution Mapping [32]. Additionally, funders today can enjoy access to a variety of automated (but not always low-cost or low-effort) impact data sources that combine data on funding patterns and project outputs and outcomes, including Dimensions.ai and Researchfish, as well as sophisticated bibliometric data sources that can help funders understand the research of their funded work in journals, policy documents and media, via resources such as Clarivate’s InCites and Web of Science, Overton, and Altmetric. However, having a plethora of RIA models and automated data sources to choose from does not automatically make RIA “easy” or “routine” for research funders.

To design a RIA, research funders face several critical decisions:

1. Which research portfolio should they assess?
2. What period of the selected portfolio should they assess? Will they assess only completed projects or include projects that are still ongoing? Are they able to factor in adequate lag time for impacts to accrue, or are assessment commissioners (often not the impact evaluators themselves) requesting a more immediate analysis?
3. What model/framework should they use to design their assessment?
4. What methods of data collection should they use?
5. Who will participate in the assessment?
6. What results should they present to the assessment commissioner?
7. What will be the next steps for the assessment? Will evaluators provide feedback on what worked and what did not work for the assessment commissioner? Are they able to incorporate changes into the funders’ evaluation practices for future assessments?

Often, the existing literature on RIA models has fallen short of providing specific implementation advice that funders can use to consider how best to approach the critical decisions laid out above. In order to understand which research funders are undertaking RIAs, and how they are thinking about these critical decisions as they design their assessments, we designed a literature review study to determine (1) which research funders have performed RIAs of their research portfolios to date; (2) how funders have designed their assessments, including the models and tools they have used; (3) what challenges to and facilitators of success have funders found when adopting the RIA model to their own portfolio; and (4) who participates in the assessments.

## Methods

### Literature sources

A literature review was conducted to assess the landscape of biomedical and health research funder impact assessments that have been performed to date. The question “how do biomedical research funders assess the impact of their investments, and what frameworks, methods, and outcomes do they report” was used to guide the review. Five published databases (Ovid MEDLINE, Ovid Embase, Ovid Global Health, Scopus and Web of Science) along with grey literature derived from Google Scholar, London School of Hygiene & Tropical Medicine (LSHTM) Theses, ProQuest Dissertations & Theses Global, the United States National Library of Medicine Bookshelf, and papers identified by key contacts were used to query literature from 2014 to 2021 for the review. Additionally, the journals *Health Research Policy and Systems*, *Research Evaluation* and *Implementation Science* were hand-searched for relevant articles published between 2014 and 2021. A previous review by Hanney et al. in 2017 [18] used a similar search strategy to identify articles featuring impact assessments of multi-project research programmes from literature published from 1990 to 2014; our review sought to capture new literature in this space, albeit with a slightly different focus on research funder-led impact assessments.

### Search strategy

A broad keyword searching strategy was performed using term domains such as *assessing impacts*, *science of science research*, *utilizing research*, *translating research and knowledge*, *citation mapping*, *bibliometrics* and *grant funding.* Search terms were developed in consultation with an LSHTM librarian and subject matter experts, and through a review of search strategies seen in the current literature. To ensure the original search was broadly capturing as many relevant articles as possible, biomedical and health-related terms were not included in the search criteria. However, one of the first steps in the article review process was to ensure that the article focused on an element of biomedical or health research funding.

The publications “Evaluation of the impact of National Breast Cancer Foundation-funded research” by Donovan et al. [33], and “Measuring research impact: a large cancer research funding programme in Australia” by Bowden et al. [34], were identified as key example papers and used to test the search syntax for each database to ensure publication retrieval was working as intended. These publications were considered key example papers because they were well known to the authors and covered key elements of the search: (1) they were published within the search time frame; (2) their primary focus was a RIA within an identifiable RIA framework described in the “Methods” section; (3) the RIA covered a defined projects portfolio for a specified research funder; and (4) they reported on project outputs and impacts. A series of Medical Subject Headings (MeSH) terms and Web of Science categories were applied at the end of each search to refine result lists. These terms related to *programme impact*, *evaluation research*, *cost–benefit analysis* and *biomedical research* and were identified by reviewing the MeSH term lists for five example papers [33–37]. The Hanney et al. 2017 review used a similar search strategy, with comparable keywords and MeSH terms [18]. A comprehensive grey literature search strategy was developed to capture any relevant white papers and funder reports located outside bibliometric databases. Full search strings are available for both the database and grey literature search strategies in Additional file 1: Table S1.

### Inclusion and exclusion criteria

Articles were included if they were published between 2014 and 2021, were English-language, and were a full-text primary research article or report. Due to our interest in what funders were doing in the RIA space, we included assessments that had been performed by external evaluators or by the funder themselves. We restricted articles based on funding mechanisms, unit of assessment and outcomes studied. The following hierarchy shows the logic applied during the review process (see Table 1 for full details):

- First, included articles needed to focus on impact assessment, specifically looking at what impact the work had on research, policy, practice and/or broader health and society.
- Secondly, the article needed to focus on a specific funding scheme (i.e. the funder was named within the article), and the unit of analysis needed to be a funding portfolio (i.e. at least two or more grants or projects). There were many articles captured within the search criteria that looked at the impacts created by research funding but did not evaluate the outputs and impacts of individual research projects. We excluded articles that looked at the impact of research funding on individuals, networks and collaborations, institutes or departments, or otherwise did not focus on the impacts of project- or grant-based funded research, as we felt these represented different types of impact assessments that focused more on researcher/departmental productivity and capacity-building, and have been featured more prominently in the literature already.
- Finally, the outcomes of the assessment needed to focus on the impact of the research funded, and not just on funding patterns over time, or funder processes such as grant applications or peer review. Reported impacts could include citations/publications; case studies/research accomplishments; collaborations/networks; capacity-building/career advancement; future funding (applied or secured) or research targeting; media citations/presentations; products/research tools/patents/drugs; clinical practice/policy/commission memberships; other broader health/economic/societal impacts; and/or return-on-investment (ROI) studies. For the purposes of this analysis, broad or long-term impacts of research were defined as: (1) impacts to clinical policy or practice; (2) other broad health/economic/societal impacts; or (3) ROI studies. We included impacts to clinical policy or practice as part of our definition of broad or long-term impacts of research, for two primary reasons: (1) we were interested in RIAs that looked at measuring the effect of funded research projects on patients and the public, and impacts to clinical policy or practice are one way to measure research impacts that have the potential to directly affect patients and the public [19]; and (2) broader health, economic and societal downstream impacts of research are among the hardest research impacts to identify, measure and correctly attribute to individual research projects/research portfolios, and we wanted to ensure that we were not being overly restrictive in our exclusion criteria [19, 38]. Examples of excluded articles were general bibliometric trends for a research field, assessments looking at the impact of funding on individuals’ research productivity [39–41], ability to procure future funding [42], or career trajectories [43]; assessments looking at the impact of funding of networks [44] or collaborative research or practice organizations [45, 46]; and assessments looking at institutes or departments such as medical schools [47, 48]. These restrictions were made carefully, to ensure that included articles focused exclusively on the review question: *how do funders assess the impact of their investments?*

**Table 1:** Inclusion/exclusion criteria for reviewed articles

	Inclusion criteria	Exclusion criteria
Initial data check	Published between 2014 and 2021	Published before 2014
	Written in English	Language other than English
	Full-text primary research, reports, systematic reviews	Letters, editorials, conference abstracts
Biomedical health	Primary focus on health	No focus on health
Impact assessment	Includes impact assessment/analysis Use of impact framework or discussion of theory preferred but not required To be included, impact analysis should include evidence of the impact on research, policy, practice and/or broader health and society	Limited/no impact assessment/analysis, i.e. the article focuses more on outputs or monitoring Articles that focused on bibliometrics only were excluded
Funding programme	Refers to a specific funding portfolio/project/grant/programme for a discrete research activity and mentions the funding body	No mention of funding portfolio/project/grant/programme OR mentioned a funding programme that looked at the impact of research funding on individuals, networks and collaborations, institutes or departments, or otherwise did not focus on the impacts of project- or grant-based funded research
Assessment focus	A portfolio of grants, projects or contracts is assessed	Only one grant/project is assessed OR did not look at the impacts of the funded research programme but rather patterns of funding over time or funder processes such as grant applications or peer review

Article searching was performed iteratively between April 2021 and February 2022, and article review, coding and analysis occurred between May 2021 and March 2022.

### Deduplication of citations

**Fig. 1:**
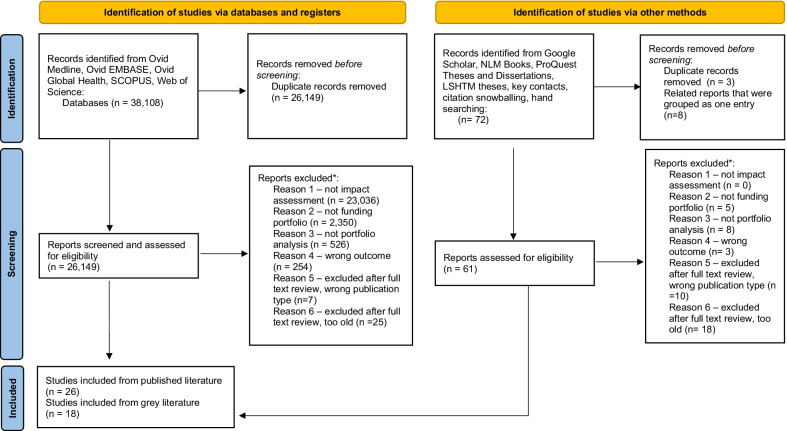
PRISMA [Preferred Reporting Items for Systematic Reviews and Meta-Analyses] 2020 flow diagram for new systematic reviews which included searches of databases, registers and other sources [98]. For more information, visit: http://www.prisma-statement.org/

Results from the five published literature databases searched were compiled in EndNote [49], totalling 38,108 citations. A thorough deduplication was conducted in EndNote, using both the “find duplicates” button and a manual review of the citations, and 37,427 articles remained. Citations were then imported into Rayyan [50] and further deduplicated using Rayyan’s duplicates function. Citations were accepted as a duplicate if the percent duplicate assigned by Rayyan was 95% or greater; those with less than 95% duplication scores were kept as unique articles. Following this process 26,149 citations remained. Seventy-two articles were gathered during the grey literature search; one duplicate article was detected, for a total of 71 grey literature articles. Figure 1 provides an overview of the article identification, screening, and inclusion/exclusion process.

### Coding and data extraction

The published database and grey literature articles were reviewed separately and then combined for data extraction. All articles were reviewed in Rayyan by title, and abstract when needed, and coded as “included”, “excluded” or “maybe” against the inclusion/exclusion criteria listed in Table 1. “Maybe” articles were screened again via abstract, and the full text was consulted, when necessary, to make a final decision on coding. Keyword spot-checking was performed for terms such as “impact”, “bibliometrics”, “funder and “payback”, to ensure that no relevant articles were overlooked. All articles were coded by RA and KO, and included articles were screened jointly to ensure that both reviewers agreed on the final selection of articles.

After an extensive review, 99.9% (26,122) of articles found via database searching and 76.4% [55] of grey literature articles were excluded. Articles may have been excluded because they were not related to biomedical health, were not related to a funding portfolio, did not perform a RIA, or performed a bibliometrics-only-focused assessment. A high rate of exclusions was expected due to the broad keyword strategy, which was designed to be as inclusive as possible to all potentially relevant literature. A final data set of 44 articles remained after a full-text screen. In instances where both a peer-reviewed article and corresponding white paper were retrieved for the same project, the published article was taken as the primary paper for the review, and any additional relevant information from the white paper was included with the published paper.

During the data extraction phase, all articles were coded on the following list of items: author country, study country, funder name, whether the article was commissioned by the funder, the funder’s health specialty, funder type (public, academic, charity, nongovernmental organization [NGO], mixed funding type), size of portfolio being assessed, portfolio funding mechanism, whether impact assessment frameworks were discussed and which impact assessment framework was used (if any), reporting time frame of the portfolio, whether the portfolio’s projects were completed or still ongoing, methods for assessing impact and their data sources, impact categories reported, whether funding dollars for portfolio projects were included, whether broader impacts were discussed, motivations for starting the assessment, goals/aims for the assessment, whether the assessment was used to make any programme changes, and whether end users were included in the assessment. To complete data extraction, we reviewed the full article, author affiliations and funding acknowledgements.

## Results

Overall, 44 papers were found to meet the review criteria. Five papers were included because they largely met the inclusion and exclusion criteria of our review, and we felt we could not otherwise exclude them. However, due to their more narrative style, we were unable to fully code them as we did for the rest of the data set. When this happened, we coded them as “not possible to code” within our data [51–55]. These papers represented two papers about the United States National Institutes of Health (NIH) [52, 53], one about Australian funding [51], one about the European Commission [54], and one about the International Development Research Centre [55]. A summary of results is provided in Table 2, and an Additional file 2 shows all included articles coded in detail.

**Table 2:** Key metrics of included articles

Metric	Total (*n*)	Total (%)
Included articles	44	100
Types of frameworks used^a^		
Any framework used	26	59.1
No framework used	18	40.1
Payback Framework	9	20.5
Own creation, not named	5	11.4
CAHS framework	3	6.8
Alberta Innovates Research Impact and Innovation Framework	1	2.3
AHRQ impact factor	1	2.3
APHIR Evaluation Framework	1	2.3
Australian Research Council Pilot Impact Assessment Framework	1	2.3
Complexity theory	1	2.3
Context matters framework	1	2.3
Contribution mapping	1	2.3
Di Ruggiero et al. field-building framework	1	2.3
FAIT framework	1	2.3
Intervention mixed-methods framework	1	2.3
Type of methods used for assessment^a^		
Bibliometrics	36	81.8
Desk-based research/documentary analysis	35	79.5
Survey	18	40.1
Semi-structured interviews	17	38.6
Case-study analysis/structured narrative	11	25.0
Field visits	4	9.1
Peer/panel review	4	9.1
Scoring of projects	2	4.5
Workshops	2	4.5
Cost–benefit analysis	1	2.3
Complexity theory	1	2.3
Data envelopment analysis (DEA)	1	2.3
Delphi survey	1	2.3
FAIT scorecard	1	2.3
Factor analysis	1	2.3
Internal rate of return/ROI	1	2.3
Landscape analysis	1	2.3
Network analysis	1	2.3
Stakeholder consultation	1	2.3
Types of impacts reported^a^		
Clinical practice/policy/commissions	36	81.8
Citations/publications	31	70.5
Products/research tools/patents/drugs	29	65.9
Broader health/economic/societal impacts	27	61.4
Media/presentations	25	56.8
Capacity-building/career advancement	24	54.5
Case studies/research accomplishments	22	50.0
Future funding (applied or secured) or targeting	20	45.5
Collaborations/networks	18	40.9
ROI studies	8	18.2
Number of total impacts reported		
1–4	9	20.5
1	0	0.0
2	0	0.0
3	4	9.1
4	5	11.4
5–9	30	68.2
5	7	15.9
6	2	4.5
7	6	13.6
8	12	27.3
9	3	6.8

^a^Totals could add up to more than 100%

### Which funders have published RIAs of their portfolios?

Included articles featured over 30 funders from nine countries (Australia, Brazil, Canada, Ireland, Malaysia, the Netherlands, Norway, the United Kingdom and the United States) and one country block (the European Union [EU]). Authors from the United Kingdom authored the most articles (12), followed by authors residing in the United States (8), Australia (6) and Canada (6). EU funding programmes were detailed in four articles. Papers from the United Kingdom and Australia were featured consistently across the review period, papers from the EU featured more heavily at the beginning of the review period (2014–2017), and papers from Canada and the United States featured more heavily at the end of the review period (2017–2021).

Funders were classified into six types: academic, charity, public, NGO, mixed (the assessment covered funding from more than one type of funder), and other funders (not otherwise specified in the article). Most assessments covered public funders (33, 75.0%). One article featured an academic funder [56], three articles featured charity funders [33, 57, 58], four articles featured NGO funders [55, 59–61], five articles featured a funding portfolio of mixed funder types [34, 35, 62–64], and two articles did not specify a funder type for the funding portfolio they assessed [51, 65].

The size of both the funder being assessed (in terms of the funder’s annual research budget) and the funding portfolio (the number of projects included in the paper’s RIA) being assessed varied greatly. Included papers covered high-profile research funders such as the United Kingdom Medical Research Council (MRC) [66] and the Australian National Health and Medical Research Council (NHMRC) [67], as well as smaller, disease- or topic-focused funders such as the Community Pharmacy Foundation [59] based in the United States and Marie Curie [58] based in the United Kingdom. Notably, included papers may have covered a higher-profile funder such as the United States Agency for International Development (USAID) but focused on a small subset of projects (the United States President’s Emergency Plan for AIDS Relief [PEPFAR] HIV/AIDS implementation science awards [68], of which 10 projects were profiled), or could have covered a smaller funder, such as the Community Pharmacy Foundation, which analysed 58 projects. The four most frequently observed funders in our portfolio were the United Kingdom National Institute for Health Research (NIHR) (5) [69–73], the European Commission (4) [54, 74–76], MRC (3) [64, 66, 77] and NIH (3) [52, 53, 78].

It is worth noting that the capacity to perform RIAs of funder portfolios is likely concentrated among a small community: five first authors were attributed to two review papers each (Angulo-Tuesta [79, 80], De Jongh [81, 82], Glover [35, 62], Guthrie [71, 72] and Welch [52, 53]), and many article collaborators appeared across multiple papers within the review.

### Why are funders undertaking an impact assessment of their portfolio, and what goals do they have for their assessment?

For some funders, the current assessment had been recommended after the completion of a previous impact assessment, which was not always completed by the current evaluators. Both Guthrie et al. and Rollins et al. noted that their evaluations were the direct result of recommendations made by other evaluators who had previously assessed their funders’ research portfolios and recommended longer-term, more comprehensive assessments [56, 71]. Other assessments represented an opportunity to utilize previous investments that the funder had made in project monitoring and evaluation data systems. In Ireland, Curran and Barret noted that the Health Research Board had set up a monitoring and evaluation framework earlier in the decade and now had 10 years of data ready for a comprehensive, multiyear impact assessment [83]. Some assessments were directly commissioned by funders to explore a methodology that the authors were experienced in; for example, Kamenetzky et al. had previous experience analysing Research Excellence Framework (REF) 2014 data and were later asked by the director of research and development at the United Kingdom NIHR to explore the impacts of NIHR’s funded medical research portfolio using data from REF 2014 case studies [73]. In the case of the MRC, their 2017 impact report represented an important evolution of their previous assessment efforts using Researchfish [66]. The 2017 report utilized the updated Researchfish^®^18 data set and included the new revised set of United Kingdom Research Councils common indicators for research assessment. Finally, some funders commissioned impact assessment studies to explore a specific dimension of their portfolio’s impact: Fun et al. in Malaysia examined how RIA could be incorporated into the research priority-setting and funding allocation process [84], and Pelletier et al. in Canada explored how RIA could be used to understand a research programme’s field-building reach [61].

### Goals for the assessment

After looking at why research funders commissioned a RIA, we then reviewed the diverse and wide-ranging goals funders presented for their assessment. Some funders stated that they were interested in looking generally at the types of impact arising from their research portfolio (Brazilian Ministry of Health [79]), demonstrating accountability to the taxpayers who had funded their research (Alberta Innovates [85]), and championing the role of their funding within the larger research community (the United Kingdom Association of Medical Research Charities (AMRC) [57]). AMRC shared that “a core part of our organisational strategy is to champion the unique voice of the medical research charity sector” and that they had a unique ability to collate impacts across a diverse set of medical research charities and highlight the important role that charity-funded research could play within the broader medical research field [57]. For some funders, an important programme milestone represented an opportunity and/or a requirement to review the impacts and efficiency of the research programme. ZonMw (the Netherlands Organisation for Health Research and Development) commissioned the external evaluators the Technopolis Group to perform an interim evaluation of their programme 5 years after the programme launch [81], and Isetts et al. shared that “[t]he Community Pharmacy Foundation utilized the 10-year grant completion (2004–2014) milepost as an opportunity to conduct this program evaluation” [59].

Other funders were interested in exploring the role their research funding programme may have had in a funded project’s ability to achieve impact. Ayenew et al. at the United States Centers for Disease Control and Prevention (CDC) examined the role their Achieving Public Health Impact through Research (APHIR) contract mechanism played in helping individual projects achieve their intended project outcomes [86], and Javorka et al. sought to determine the added value of a concordat (joint funding agreement) between the United Kingdom MRC and Department for International Development (DFID) during 2013–2018 [77]. Further, Bleecker et al. and Javorka et al. were interested in examining how their funding programmes not only created impact but supported the dissemination of the resulting research [55, 77].

In some cases, funders shared that the goal for their assessment was to provide sufficient evidence of impact to determine whether the programme should be renewed for future funding [59] or design for future iterations of the funding programme [75]. Boulding et al. looked in depth at the NIHR’s public health research portfolio to qualitatively examine researcher perspectives on what counts as impact and impact pathways, and how research is disseminated [69]. Marie Curie shared that a key goal for their analysis was to identify gaps in the funding/evidence landscape for palliative and end-of-life care [58].

Finally, two assessments were not led by funders themselves, but designed to explore new methodologies for impact assessment using a funded research portfolio as a case study for their analysis. Mulligan developed a PhD thesis to explore how a methodology called data envelopment analysis could be used to examine the comparative efficiencies and impacts of individual malaria research projects funded by the MRC [64]. In Australia, Newson et al. trialled a novel impact assessment methodology that traced impact both forward from individual research projects and backward from childhood obesity prevention policies in New South Wales from 2000 to 2015 [51].

### How are funders designing their assessments? How are they selecting which portfolio to assess, and which frameworks, methods and data to utilize?

#### Size of the portfolio being assessed

Included papers covered a range of portfolio sizes. Twelve papers covered small impact assessments (less than 50 projects from a funder were included in the assessment); 11 papers covered medium impact assessments (51 to 150 projects from a funder were included in the assessment); seven papers covered large impact assessments (151 to 500 projects from a funder were included in the assessment); and 12 papers covered extra-large impact assessments (over 500 projects from a funder were included in the assessment). The smallest portfolio assessed within the review was an Australian study that looked at two global health projects, and the largest portfolio assessed within the review was a grey literature report from the United Kingdom’s AMRC which covered 10,579 projects. Two papers were not possible to code as they did not reveal any information about the size of the portfolio being addressed.

#### Health topic area of the portfolio being assessed

The content of included research portfolios varied greatly. Six papers included research portfolios focused on global health and/or low- or middle-income country (LMIC) topics, and many of these papers mentioned efforts to survey or visit in-country collaborators during the RIA process [75, 77, 82]. Additional papers may have covered portfolios with international collaborators, but these were not well specified. Eleven papers did not specify any health topic areas covered by their research portfolios. Topics in papers that did specify the area covered included cancer (3); clinical trials or the Clinical and Translational Science Awards (CTSA) Program (3); child health (2); food systems research (2); health prevention (2); pharmacy/pharmacotherapy (2); biomedical research infrastructure (1); environmental health (1); family well-being (1); heart, blood and lung (1); the Health Technology Assessment (HTA) Programme (1); implementation science (1); infrastructure research (1); musculoskeletal (1); neuroscience (1) and terminal illness (1). Three papers listed several health topics covered by their research portfolios and were coded as “many”.

#### Unit of portfolio being assessed

Most included articles (33, 75.0%) featured impact assessments that dealt with funding portfolios based at the project or grant level. Articles addressing other types of research funding included the following: two articles were large ROI studies which looked at all research funding in a particular sector (from multiple funders) by total research spend across funders, not at outcomes generated at the individual project level by funder [35, 62]; one article examined the impact of Cochrane reviews themselves (arising from previous NIHR project funding) [70]; two articles examined prepared case studies of funded projects [73, 87]; one article examined the collective impact of all Marie Curie palliative and end-of-life care funding, including research projects but also research centres, programme leads, hospices and fellowship funding [58]; one article examined the scientometric impact of researchers funded by European Research Council projects [76]; two studies examined the impact of the NIH CTSA Program hubs, where the unit of analysis was a CTSA hub (which necessarily meant they had been the recipient of an NIH-funded award) [52, 53]; one study examined the impact of European biomedical research infrastructures in the fight against COVID-19 [54], where the unit of analysis was funded infrastructures but the project/award mechanisms were not well specified; and two studies did not provide enough information to determine the unit of portfolio being assessed [55, 88].

#### Project completion status and timeline for assessment

Thirteen articles (29.5%) included completed projects only and another 13 articles included both projects that were completed and some that were still ongoing. Eighteen articles (40.9%) did not include enough information about the included projects to determine project completion status. Most projects (41, 93.2%) included the years the projects were funded (i.e. projects in this assessment were funded during 2013–2017); however, it was not possible to systematically report on the lag time funders may have incorporated into the assessment, as articles did not routinely report on the time between the end of research funding and the beginning of data analysis.

#### Frameworks

Twenty-six articles (59.1%) cited the use of a framework for their study design, and 18 (40.9%) did not mention the use of a framework at all. Twelve named RIA frameworks were cited across the review, and the Payback Framework was the most frequently cited (9 papers, 20.5%). The Payback Framework was used by public funders, mixed funders, charity funders and academic funders. One named framework, the APHIR Evaluation Framework [86], was developed by Ayenew et al. (a public funder) during the course of preparations for their article, and described therein. All other named frameworks were cited in the literature prior to the articles mentioning them. Five articles, all from public funders [67, 70, 74, 89, 90], developed their own (unnamed) impact framework for their analysis, often adapted from a leading framework such as the Payback [23] or CAHS [24] frameworks. Another five articles (spanning public, NGO and mixed funders) used a more general methodology/framework that differed from the traditional impact frameworks but had been described elsewhere in the literature: Complexity Theory [55], Context Matters Framework [53], Contribution Mapping [63], Di Ruggiero et al. field-building framework [61] and a general intervention mixed-methods framework [52].

Articles that did not mention the use of an impact framework in guiding their analysis covered all funder types. Among charity funders, two out of the three funders did not use an impact framework (66.7%); among public funders, 12 out of 33 funders did not use an impact framework (36.4%); among NGO funders, one out of five did not use an impact framework (20.0%); among mixed funders, half—or two out of four funders—did not use an impact framework (50.0%); and among other/unspecified funders, also half of funders (one out of two) did not use an impact framework. Similarly, articles that did not mention the use of an impact framework also covered project portfolios of all sizes. Compared with articles examining small or medium-sized portfolios (under 150 projects assessed in total), articles examining large or extra-large portfolios (greater than 151 projects assessed in total) were more likely to report not using an impact framework. Over half of articles assessing large and extra-large portfolios (52.6%) did not report the use of an impact framework, compared with 30.4% of articles assessing small or medium-sized portfolios. One of the two articles that did not mention portfolio size did not use an impact framework.

#### Methods

Methods were coded, where possible, to the approaches and methods detailed in the Boaz et al. 2009 article, “Assessing the impact of research on policy: a literature review” [91]. Nineteen methods were recorded, and the most common methods observed were bibliometrics (36); desk-based research/documentary analysis (35); survey (18); semi-structured interviews (17); and case-study analysis (11). Thirty-three (75.0%) of 44 articles were coded as mixed-methods studies, meaning that they involved the use of both quantitative and qualitative methods. Only seven articles (15.9%) used only one type of methodology (i.e. documentary analysis only) to perform their analysis.

Articles citing documentary analysis typically analysed project documentation such as project applications, annual reports, end-of grant reports, or other such administrative or financial data about projects. For most projects, this information was provided by the funder; however, eight projects performed documentary analysis of existing Researchfish data for their portfolios [57, 58, 64, 66, 69, 71, 77, 85]. Among the 18 projects that conducted surveys to gather information on project outputs, outcomes and impacts, surveys were typically sent to project leads, who were described as principal investigators (PIs), grantees or contracting officer/project representatives. In two instances, surveys were used to gather viewpoints on stakeholder impressions of research relevance [84] and to ask Cochrane Review Groups staff about their views on the impacts of funded Cochrane reviews [70]. Semi-structured interviews were typically used in two ways: (1) to talk more in depth with PIs or project leads about their views on what factors contributed to project impacts, or (2) to capture viewpoints on project impacts from other stakeholders or key informants. Sometimes, funder assessments used interviews to learn why PIs had been motivated to apply to their funding programme [78]. Key informant interviews were also occasionally used to gather background information about the research programme so that a survey could then be developed and fielded more widely to project representatives. In other examples, the interview or survey responses collected became the basis of future case studies. Bibliometric analysis was widely used to gather publications that arose from funded research, determine their relative impact, and determine whether they had been cited in guidelines or policy documents. Databases utilized included Altmetric, Clarivate Analytics, Google Scholar, Scopus and Web of Science.

Eleven studies used case-study analysis to prepare in-depth reviews of a selection of projects. These case studies typically were prepared following interviews or surveys with PIs, and sometimes other members of the project team, as well as a review of the project documentation and outputs. Authors sometimes performed additional analyses on the survey results, interview transcripts or case studies themselves. Cohen et al. used the case studies to develop two- to three-page “impact assessment summaries” on a sample of the case studies that were then reviewed by an expert panel who assessed the impact summaries on four dimensions of impact: “corroboration, attribution, reach, and importance” [67]. Guthrie et al. qualitatively coded case studies “to identify the key impact mechanisms associated with HTA research as well as success factors, i.e. the things that support the successful translation and implementation of the findings of HTA research” [71], and Welch et al. used thematic coding to understand overarching themes in survey results and interview transcripts [52, 53]. In some instances, authors prepared narrative summaries of a project’s impact, like a case study, but called them different things. For example, Curran and Barret used “impact narratives” [83] and Kok et al. prepared “process summaries” [63] to highlight how projects transitioned from formulating results and producing knowledge to disseminating and utilizing the knowledge. Finally, Bleecker et al. used previous evaluation reports prepared by different authors to create a secondary thematic content analysis of the original materials [55]. This content analysis was used to organize findings by four Complexity Theory constructs: “emergence, unpredictability, contradiction, and self-organization” [55].

Other methods utilized included (1) expert workshops or consensus meetings to discuss case studies and impact pathways [69, 71]; (2) project scoring [68] using methodology from Kwan et al. [92], project scoring using the FAIT scorecard narrative and review method [65], project scoring using the Agency for Healthcare Research and Quality (AHRQ) impact scoring methodology [59], or expert review panels to determine whether projects produced statistically significant changes in project outcomes [67]; (3) assessing the relevance of the project to the thematic priority areas [82]; and (4) determining the impact of journal publications through expert review [76]. Some articles employed literature reviews [51, 60, 77, 84] or landscape analyses [61]. Fun et al. explored the research priority-setting processes of stakeholders using the Child Health and Nutrition Research Initiative methodology as part of a study to determine stakeholder values around research priority-setting [84]. Three studies used the internal rate of return to determine research returns [35, 62, 72], and one study used cost–benefit analysis [65]. Mulligan piloted data envelopment analysis to understand the relative efficiency of research projects in a portfolio [64]. Tsey et al. used coauthor network analysis of peer-reviewed publications of programme-funded research [87] to prepare a case study of a research programme. Cochrane et al. used an online focus group as well as a conference presentation for workshopping cross-cutting findings of prepared case studies in preparation for a final evaluation report [74]. Welch et al. employed factor analysis “to construct the composite measure of a hub’s prior experience with metric-based performance improvement” [53]. Finally, projects with international collaborators utilized field visits [77] or site visits [75] to assess on-the-ground impacts in collaborator countries.

It is important to note that the methods sections of included articles varied greatly in terms of detail. Some articles included a brief line or two about methods, whereas other articles walked through methods used in detail. Understanding the reasoning behind authors’ choice of certain methods was not always possible due to a lack of information in some articles. Furthermore, some studies were built upon data sets collected in previous evaluations (that were not included in this review), and a full description of these data sets was not always provided.

#### Reported impacts

Articles were coded by the types of impact categories reported. Ten overarching research impact categories were developed based on the most common impacts seen within the articles: citations; case studies/research accomplishments; collaborations/networks; capacity-building/career advancement; future funding (applied or secured) or research targeting; media/presentations; products, research tools, patents or drugs; clinical practice/policy; broader health/economic/societal impacts; and ROI studies. The most frequently reported impact categories were clinical practice/policy impacts (36, 81.8%); citations (31, 70.5%); products, research tools, patents or drugs (29, 65.9%); broader health/economic/societal impacts (27, 61.4%); media/presentations (25, 56.8%); and capacity-building (24, 54.5%). Only eight studies reported ROI measures. Thirty-nine studies (all but the five studies that could not be fully coded) reported some impacts, and the average number of impact categories a study reported on was six.

#### Reported programme changes following assessment

Articles were reviewed to determine whether authors provided any details on potential programme changes that resulted from the assessment. Thirty-one (70.5%) articles did not mention how the assessment would be used to create future programme changes. The remaining 13 (29.5%) articles provided recommendations for the funder to consider or noted programme changes that were beginning following the completion of the assessment. For example, Angulo-Tuesta et al. noted that their assessment provided recommendations on how to strengthen the Brazilian government research system [79], and Ayenew et al. noted that their assessment had encouraged CDC to develop an ongoing project monitoring system for the APHIR programme [86]. Waterhouse et al. shared that both funders included in their analysis planned to incorporate their survey into every end-of-grant report and conduct follow-up surveys 6 months and 1 year out from the end of a project [90]. Bradley-Dexter et al. identified a gap in evaluation capacity among their grantees during the programme and worked to both develop evaluation resources and engage outside evaluation experts that could aid their grantees in ROI analyses and evaluations of their work’s influence on policy [88]. Other programme changes or follow-ups noted included recommendations to explore greater collaborations between investigators and academics [59]; provide more guidance to applicants on how to complete “pathways to impact statements” at the project proposal stage, anticipating that it may help researchers better set their projects up for impact generation [77]; assign a dedicated monitoring and evaluation point person within the programme to coordinate evaluation efforts [75]; and require grantees to report the potential influence and public benefits of their award [89].

### What challenges to and/or facilitators of success have funders observed when implementing impact assessments of their research portfolios?

In addition to reporting on the impacts of their research investments, some funders offered advice about factors that contributed to successful assessments, as well as challenges that arose during the assessment process. Generally, funders that made end-of-grant impact reporting required [34] and funders who had previously invested in evaluation and monitoring systems [78, 83, 88] found these processes to be helpful in generating the kinds of data needed to perform the impact assessment. The Public Health Agency of Canada’s Innovation Strategy programme provided expert evaluation support to projects to help with “return on investment analysis, the evaluation of policy influence, and one-on-one support for tailored intervention plans” [88]. While this level of evaluation support goes above and beyond what was typically provided by funders in this portfolio, the authors noted that “incorporating evaluation expertise in each funded project ensured that interventions were tested to better understand the impact of their activities, which added to the evidence base on effective approaches” [88].

On the flip side, funders faced numerous challenges when performing their impact assessments. These challenges ranged from (1) the struggle to balance the comprehensiveness of an evaluation with overall feasibility of performing such an evaluation [67]; (2) the problem of having limited funder data collection systems and needing to offer an alternative data collection method instead, such as interviews [55]; (3) the desire to use complex indicators such as citation indicators to measure impact but not having enough data, time and resources to incorporate this methodology [79], or feeling like the current methodology they were intending to use was not developed enough and more research would be needed, such as incorporating complex ROI metrics [70]. Additionally, several challenges dealt with the perspective of time within the evaluation: (4) evaluators wanted to assess impact over a long time frame but faced time, money and/or staffing constraints to do so [55]; (5) evaluators were hoping to assess long-term impact in the future, but their current programme assessment was performed too early in the programme’s lifespan to do so [60, 86]; and (6) evaluators felt they might have been better able to capture impacts if the evaluation had been performed prospectively instead of retrospectively [55, 79].

### Who participates in funder assessments of research portfolios?

Sixteen of the 44 articles (36.4%) included mention of surveying or interviewing end users as part of the evaluation process, and only one article mentioned the inclusion of members of the public in their assessment [71]. Some examples of end users (also known as stakeholders) featured in assessments included academics topical and/or international experts [60, 61, 71, 74, 77]; decision-makers or government officials [51, 60, 71, 74, 77, 82]; donors, NGOs, charities and/or other funders [60, 61, 71, 74, 75, 77]; guideline developers [70, 72]; industry representatives/product development partnerships [23, 74]; and unsuccessful PI applicants [81]. Specific descriptions of included end users were not always present in the articles; sometimes these groups were just referred to generally as “end users” or “stakeholders” [63, 72]. Furthermore, internal programme staff were sometimes included in assessments as survey participants or key informants [60, 70, 71, 75, 77, 81, 82].

## Discussion

Within the field of RIA, there have been several published reviews on the value of RIA and available RIA frameworks [9, 10, 18–21], and formative guidelines from the International School on Research Impact Assessment (ISRIA) on how to produce an effective RIA [8] have recently been published. Impact assessment can be performed by a variety of actors including researchers, universities and funders, and while previous work in this field has often grouped these actors together, it is important to emphasize that the needs and goals for impact evaluation for the groups may be quite different [22]. The Hanney et al. 2017 review, “The impact on healthcare, policy and practice from 36 multi-project research programmes: findings from two reviews”, was formative to our work, as it is one of the first reviews to look at the findings of individual RIA studies collectively to determine whether impacts were created by multi-project research programmes and how the included studies approached RIA, using literature published during 1990–2014 [18]. We are particularly interested in what *research funders* are doing in this space and sought to perform a comparable study looking at literature from funders from 2014–2021. Surveying the landscape of research funders performing RIAs in peer-reviewed or grey literature publications is critical to understanding which funders are already engaged with RIA, how RIAs are currently being designed by funders, the key facilitators of success and barriers to implementation for funders, and who is participating in RIAs.

This review provides a first global overview of RIA among health funders, and has revealed a growing culture of RIA among funders, with several diverse funders performing RIA on their research portfolios. The 44 included studies featured over 30 funders, and these funders generally came from countries with a demonstrated record of impact literature over the years (i.e. the United Kingdom, Australia, Canada and the United States). Importantly, funders currently engaging with RIA represent funders of all sizes and funder types (academic, charity, NGO, public and mixed funders). These findings accord with those of Hanney et al., who found a similarly diverse mix of funder types, sizes and locations in their reviews [18]. Over half of funders (59.1%) included in our review used a RIA framework to guide their assessment, and a wide variety of methods were employed to capture the impacts of funded projects. The proportion of funders using a named conceptual framework in our review is like that of Hanney et al., which found that 55.6% of included studies reported using a named conceptual framework [18]. Some of the individual frameworks used by studies in the reviews differed, however, and the Research Impact Framework, Banzi research impact model and Becker Medical Library model, used by studies within the Hanney et al. review [18], were not used by the studies included in our review. Of note, the Research Impact Framework was designed by academics for academics [18], which may help explain why it was not found in our review of impact assessments performed by funders. In both reviews, the legacy Payback Framework was the most frequently utilized framework among included studies. Individual methods used by the studies in both reviews were comparable, with studies in both reviews using surveys, documentary review and interviews to gather evidence of impact. Hanney et al. concluded their review with a discussion about the opportunity for Researchfish, launched in 2014, to become an important standardized data source for research funders to collect impact data (albeit with the acknowledgement of Researchfish’s notable limitations around researcher reporting burden). Indeed, our review found that, since 2014, eight RIA studies have used Researchfish data to perform their assessments.

Both our study and the Hanney et al. review were concerned about issues of rigour and appropriateness of methodologies taken to identify research impacts, though we approached these issues in slightly different ways. In the Hanney et al. review, these issues were discussed as limitations to the overall review, with the authors noting that some studies relied heavily on self-reported impact data by PIs or project leaders, had limited survey response rates (if a survey was used to collect impact data), and/or had issues with the timing of the assessment performed, noting that several studies were performed too early in the programme’s lifespan to be able to adequately capture research impacts. Because the goal of our review was somewhat different—to report on *what* funders are doing, rather than what level of impact research portfolios might be expected to produce—we did not view these issues (found similarly in our review) as limitations to our review itself, but rather as limitations to the quality of the studies included. The methodological rigour of the included articles was affected by several factors: (1) lack of a reference impact assessment framework (affecting 40.9% of articles); (2) lack of information about project completion status, making it impossible to assess whether an article featured only completed research projects or included both completed and ongoing projects (affecting 40.9% of articles—of note, these were not necessarily the same articles as referenced above without a referenced RIA framework); (3) a systematic lack of information about the lag time that was incorporated into the assessment to allow for potential accumulation of research impacts affecting nearly all articles in the review; and (4) a general lack of detail about methods and data sets used in some articles.

We also reviewed article texts to determine whether authors reflected on activities or systems that facilitated a robust RIA and/or discussed challenges that made it difficult to capture relevant data for their assessment. Key facilitators observed were requiring grantees to perform end-of-grant reporting and having a robust evaluation and monitoring system set up to track grantee performance. Like the Hanney et al. review, many funders included in our review were embarking on the RIA process at an earlier time point and had not yet set up these systems or employed a rigorous assessment framework and methodology for reporting on impacts. Going forward, we believe it will be important to look closely at how funders operationalize, implement and reflect on RIA processes within their institutions [15], to provide practical advice for funders on how they can improve implementation of RIA processes within their institution or begin RIA for the first time. We do not believe, based on this review, that there is yet sufficiently robust evidence about the utility of RIAs to offer best practice guidance on when they should be considered useful and most needed at the funder level. More research is needed to tease out when sufficient capacity (data infrastructure, personnel, methodology/design and lag time) has been met by a funder to allow for an appropriately rigorous RIA to begin. Further still is the available evidence needed to help a funder determine when a RIA is needed versus a less intensive and shorter-term focused follow-up monitoring and evaluation report of a funding programme.

Finally, an area of particular interest for us going forward is understanding who participates in RIAs, and whose voices are necessary to best capture research impact. A little over a third of articles (36.4%) mentioned surveying end users when collecting impact data, a number replicated by Hanney et al., who found that “over a third of the studies involved interviews with stakeholders” [18]. While we anticipated that the inclusion of patients and members of the public in the impact assessment studies would be low based on prior observations by Milat et al. and others [21], we were surprised that only one included article specifically mentioned surveying patients and members of the public to obtain their views on research impact. Furthermore, the inclusion of patients and members of the public was mentioned only briefly as one of many end users surveyed within the assessment, and a focused discussion on the value of patient and public involvement to the overall assessment was lacking. Stakeholder involvement in RIAs is considered a best practice within the field [8, 12, 15] and is critical for determining how RIA can best be tailored to the needs of the patient community [21]. Because some of the funders included in this assessment are already involved in initiatives to increase patient and public involvement (PPI) in research (for example, the NIHR provides guidance for researchers on how to incorporate PPI in their research and how to evaluate the impact of PPI on their research [93]), we believe there is an opportunity for these funders to transition to incorporating PPI in their RIAs as well. If it is important for future research to be designed to be as inclusive as possible to the needs of patients, we argue that it is just as important for evaluation of these research initiatives to include the voices of patients when determining what counts as *meaningful* research impact [94]. Reed et al. argue that this approach may mean a trade-off during assessment design between preselected impact indicators that may be already collected in a systematic fashion by funders, and new impact indicators that are chosen after discussion with the relevant patient communities [22].

While we took steps to ensure that a wide variety of published and grey literature was surveyed for our literature review, it is important to acknowledge that this review had some limitations. First, although we employed a broad keyword search strategy, it is possible that we have still overlooked some relevant literature from funders, particularly in the grey literature, as the descriptions of this type of impact assessment work can fall under many titles. Future reviews may wish to survey research funders and review funder websites, to ensure that funder RIAs and any funder RIA frameworks and implementation protocols are not missing from the literature search. Second, vetting studies for inclusion/exclusion was a difficult process because of the great variety and quality of RIAs performed, and while we established a hierarchy of included studies and discussed each included article at length, it is possible that a different set of reviewers could make different determinations about which articles to include. Furthermore, some studies seemed relevant but did not present data about their funding portfolio and impact assessment in a way that allowed for data extraction and had to be excluded. Third, it is important to note that we did not judge the quality of the RIA performed within the 44 included articles, and there was a wide range of methodological rigour and comprehensiveness among included studies. Fourth, we did not require a minimum number of projects to be present in the funder’s RIA, and the scope of included RIAs varied among funders (from two case studies per funder, to over 1000 projects per funder). It is important to note that when a particular funder was included in this review, the scope of RIA within their institution may still be quite small. Still, we believe this work provides a valuable snapshot of where research funders currently are in their journey to perform RIAs. The fact that we closely aligned our study with the previous Hanney et al. review in this topic and have found similar results suggests that this work is representative of the larger field of RIAs.

## Conclusions

Despite a growing culture of RIA among funders, the prevalence of peer-reviewed publications and grey literature of funder impact assessments remains low. We encourage both the funders included in this review and others to prioritize the placement of RIAs in peer-reviewed literature or the public domain in promotion of the ISRIA’s call for creating a “community of practice”, as highlighted as step number 10 in their 10-point guidelines for an effective process of RIA [8].

We believe that increasing methodological rigour is a priority for increasing the overall utility of funder RIAs and critical for moving the field forward. We recommend that funders pay special attention to the methods sections of future RIA articles and reports, carefully detailing (1) the portfolio of research included in the analysis, featuring a description of the type of research and the unit of analysis (i.e. research project, funded researcher, programme/centre/network [67]); (2) the dates of funded projects included in the analysis along with information about whether the projects included were completed at the time of analysis or are still in progress; (3) clear dates of when the analysis took place so that a calculation of included lag time can be made; (4) careful documentation of methods, data sets and tools used to perform the analysis, including notation of whether the assessment was performed by internal funder/programme staff or external evaluators; and (5) clear documentation of any end users or members of the public included in the assessment. In general, we advise that funders prioritize RIAs that feature only completed research projects, as the inclusion of ongoing research projects muddies the assessment of potential versus realized impacts. Additionally, it is important for funders to wait after the completion of a funding programme for substantial lag time to accrue before beginning an assessment, to ensure that the assessment can have the greatest chance of capturing longer-term research impacts such as impacts on clinical practice, policy and health. While the notion of research impacts taking 17 or more years to be generated from a starting research project to a product is popular within the RIA community [95], the COVID-19 pandemic has demonstrated that immense research impacts can occur in less than a year (from sequencing of the COVID-19 virus in January 2020 to emergency use authorization of the COVID-19 vaccine for healthcare professionals in the United States in December 2020) [96, 98]. For this reason, we encourage a shorter average minimum time frame of 3 years for lag time to accrue, with notable exceptions if needed for pandemic-era RIA. We acknowledge that this may require some significant work on the part of evaluators to advocate for additional time and resources to perform these assessments, as Kamenetzky et al. have reported [15], especially in a funder culture that may value immediate demonstration of funded project results. We hope that future work to develop additional evidence around the capacity and skills a funder needs to perform a methodologically rigorous RIA will allow funders to make a more informed choice about whether their organization has the ability to perform (or contract with an external evaluator to perform) a RIA, or whether a less intensive and shorter-term focused monitoring and evaluation report of a funding programme is more appropriate for their needs and current skill set.

Additionally, we believe that funders could benefit from additional research into implementation best practices that go beyond the methodological so-called quick-fixes that have been recommended. After a funder has carefully reflected on their organization’s data capacity and evaluator skill sets needed to perform a RIA and made the decision to embark on a RIA, they face the difficult task of deciding which of the numerous frameworks, methodologies and automated impact data sets available within impact assessment literature to employ for their analysis. The current literature correctly asserts that there is no “one-size-fits-all” approach to RIA [8], which we wholeheartedly agree with; however, there may be a “happy medium” approach that offers funders more practical advice about options they can take depending on a few typical goals for assessment. At present, there is insufficient evidence to develop or support best practice in RIA framework and methodology selection for funders. More empirical evidence is needed to offer a top two to three recommended RIA frameworks and methodologies for funders wishing to demonstrate general impacts of the portfolio and a top two to three recommended RIA frameworks and methodologies for funders wishing to demonstrate how stakeholder contributions worked together to create impact and disseminate evidence. In each scenario, a recommended set of data indicators could be provided based on a range of available data. Through this process, we hope funders may be encouraged to realize that a novel funder-specific framework may not be needed for their analysis, and that they could benefit from using a more standardized framework and choosing a few additional indicators to measure their funder-specific goals. We believe that further research to help simplify the process of selecting a RIA framework will encourage more funders to adopt RIA activities in the future, contribute to a more standardized evidence base, and discourage the proliferation of only slightly different RIA frameworks that seem to have plagued the current field. Facilitating continued collaborative learning across funders and RIA experts will be important to ensure that there is community buy-in for standardizing the field of RIA and promoting and creating an open evaluation culture.

Finally, we feel that one critically important stage of RIA has been unfortunately overlooked by many of the current funder assessments in the literature—a careful reflection of the voices needed in an impact assessment to ensure that RIAs have a meaningful impact on patients and the public. While many assessments within the review featured sections on impact on practice or policy, we note that only one assessment mentioned including perspectives from patients and members of the public in their analysis. We urge funders to prioritize the collection of data regarding impacts on patients and members of the public when designing new RIAs, so that they can be sure that their research is creating the intended benefits for patients and demonstrating relevance to the general public [67]. Such data could come from research project application and end-of-grant reporting impact statements, surveys or interviews, or focus groups/review panels that include patients or members of the public. We also encourage funders and researchers to consider the involvement of patients and members of the public throughout the life cycle of the RIA—including the design phase, the measurement phase and the analysis phase. Future qualitative research to understand how patients and members of the public conceptualize “research impact” more fundamentally can help funders ensure that RIAs are designed to measure impacts that patients value, rather than just asking for their opinions on impact data that has already been collected. Overall, we believe that much opportunity for funder RIAs lies ahead.

## Supplementary Information

**Additional file 1.** Search criteria for review.

**Additional file 2.** Included papers table.

## Data Availability

Full search strings have been provided for all literature review searches performed. In the additional table provided, all included articles in the review have been provided with the original article codes used in this analysis.

## Abbreviations

AMRC: United Kingdom Association of Medical Research Charities
APHIR: Achieving Public Health Impact through Research
CAHS: Canadian Academy of Health Sciences Framework
CDC: United States Centers for Disease Control and Prevention
CHSPRA: Canadian Health Services and Policy Research Alliance
DFID: Department for International Development
FAIT: Framework to Assess the Impact from Translational Health Research
HTA: Health technology assessment
ISRIA: International School on Research Impact Assessment
LMIC: Low- and middle-income countries
MRC: United Kingdom Medical Research Council
NHMRC: Australian National Health and Medical Research Council
NIH: United States National Institutes of Health
NIHR: United Kingdom National Institute for Health and Care Research
NGO: Nongovernmental organization
PEPFAR: United States President’s Emergency Plan for AIDS Relief
REF: Research Excellence Framework
RIA: Research impact assessment
ROI: Return on investment
SIAMPI: Social Impact Assessment Methods through Productive Interactions
USAID: United States Agency for International Development
ZonMw: Netherlands Organisation for Health Research and Development
