# Supplementary material for: What funders are doing to assess the impact of their investments in health and biomedical research
Source: Health Res Policy Syst. 2022 Aug 9;20:88. doi: 10.1186/s12961-022-00888-1 (PMC9361261; doi:10.1186/s12961-022-00888-1)
Supplement: Supplementary file 1 — Additional file 1. Search criteria for review. [file 12961_2022_888_MOESM1_ESM.docx]

**Additional File 1. Search Criteria for Review**
Table S1. Search Strategy for OVID MEDLINE

| Term Domain | Search Terms |
| --- | --- |
| #1  Assessing impacts | (((assess* or eval* or analy* or frame* or compar* or review* or explor* or identif*) adj3 (impact or contribut* or payback or role or util* or outcom* or influenc* or output* or return* or effect* or productiv*) and (research* or grant* or portfol* or invest* or fund*)).ab,ti. |
| #2  Frameworks | (SIAMPI or “social impact assessment” or “productive interactions” or ASIRPA or PIPA or “participatory impact pathways analy*” or “Payback framework” or “research impact framework” or “research excellence framework” or “research assessment exercise” or “Canadian academy of health sciences” or “research quality framework” or “excellence in research Australia” or “measurement of research impact and assessment” or “Weiss logic model” or “program assessment rating tool” or “Canadian Institutes of Health Research framework” or “HTA Quebec model” or “research utilization model”).mp. |
| #3  Science of science | (“science of science” or “research on research”).ab,ti. |
| #4  Utilizing research | ((research adj3 util*) or (research adj3 us*) or (evidence adj3 pract*)).ab,ti. |
| #5  Translating research /knowledge | ((translat* adj3 research) or (translat* adj3 knowledge) or “knowledge production”).ab,ti. |
| #6  Citation mapping | (“citation mapping”).mp. or Bibliometrics/) |
| #7  (Combine) | 1 OR 2 OR 3 OR 4 OR 5 or 6 |
| #8  Grant funding | (grant* or scheme* or scholarship* or portfolio* or studentship* or award* or fellowship* or research* or return* or train* or capacity* or fund*).ab,ti. |
| #9  (Combine) | 7 AND 8 |
| 10  MESH Terms | exp Career Mobility/ or exp Cost-Benefit Analysis/ec, mt, sn or exp Health Policy/ec, sn or exp Health Services Research/ec, sn or exp Program Evaluation/ec, sn or exp Research Personnel/ec, ed, sn or exp Research Support as Topic/sn or exp Translational Medical Research/ec, sn. or exp Health Care Costs/sn or exp Capacity Building/ec, sn or exp Biomedical Research/ or Outcome Assessment, Health Care/ |
| #11  (Combine) | 9 AND 10 |
| #12  (Combine) | limit 10 to (English language and humans and yr= “2000-Current”) |

Table S2. Search Strategy for OVID EMBASE

| Term Domain | Search Terms |
| --- | --- |
| #1  Assessing impacts | (((assess* or eval* or analy* or frame* or compar* or review* or explor* or identif*) adj3 (impact or contribut* or payback or role or util* or outcom* or influenc* or output* or return* or effect* or productiv*) and (research* or grant* or portfol* or invest* or fund*)).ab,ti. |
| #2  Frameworks | (SIAMPI or “social impact assessment” or “productive interactions” or ASIRPA or PIPA or “participatory impact pathways analy*” or “Payback framework” or “research impact framework” or “research excellence framework” or “research assessment exercise” or “Canadian academy of health sciences” or “research quality framework” or “excellence in research Australia” or “measurement of research impact and assessment” or “Weiss logic model” or “program assessment rating tool” or “Canadian Institutes of Health Research framework” or “HTA Quebec model” or “research utilization model”).mp. |
| #3  Science of science | (“science of science” or “research on research”).ab,ti. |
| #4  Utilizing research | ((research adj3 util*) or (research adj3 us*) or (evidence adj3 pract*)).ab,ti. |
| #5  Translating research /knowledge | ((translat* adj3 research) or (translat* adj3 knowledge) or “knowledge production”).ab,ti. |
| #6  Citation mapping | (“citation mapping”).mp. or Bibliometrics/) |
| #7  (Combine) | 1 OR 2 OR 3 OR 4 OR 5 or 6 |
| #8  Grant funding | (grant* or scheme* or scholarship* or portfolio* or studentship* or award* or fellowship* or research* or fund*).ti. |
| #9  (Combine) | 7 AND 8 |
| 10  MESH Terms | exp program impact/ or exp translational research/ or exp medical research/ or evaluation and follow up* or Exp evaluation research or Exp evaluation study |
| #11  (Combine) | 9 AND 10 |
| #12  (Combine) | limit 10 to (English language and humans and yr= “2000-Current”) |

Table S3. Search Strategy for OVID Global Health

| Term Domain | Search Terms |
| --- | --- |
| #1  Assessing impacts | (((assess* or eval* or analy* or frame* or compar* or review* or explor* or identif*) adj3 (impact or contribut* or payback or role or util* or outcom* or influenc* or output* or return* or effect* or productiv*) and (research* or grant* or portfol* or invest* or fund*)).ab,ti. |
| #2  Frameworks | (SIAMPI or “social impact assessment” or “productive interactions” or ASIRPA or PIPA or “participatory impact pathways analy*” or “Payback framework” or “research impact framework” or “research excellence framework” or “research assessment exercise” or “Canadian academy of health sciences” or “research quality framework” or “excellence in research Australia” or “measurement of research impact and assessment” or “Weiss logic model” or “program assessment rating tool” or “Canadian Institutes of Health Research framework” or “HTA Quebec model” or “research utilization model”).mp. |
| #3  Science of science | (“science of science” or “research on research”).ab,ti. |
| #4  Utilizing research | ((research adj3 util*) or (research adj3 us*) or (evidence adj3 pract*)).ab,ti. |
| #5  Translating research /knowledge | ((translat* adj3 research) or (translat* adj3 knowledge) or “knowledge production”).ab,ti. |
| #6  Citation mapping | (“citation mapping”).mp. or Bibliometrics/) |
| #7  (Combine) | 1 OR 2 OR 3 OR 4 OR 5 or 6 |
| #8  Grant funding | (grant* or scheme* or scholarship* or portfolio* or studentship* or award* or fellowship* or research* or fund*).ti. |
| #9  (Combine) | 7 AND 8 |
| 10  MESH Terms | exp career development/ or exp interdisciplinary research/ or exp medical research/ or exp “diffusion of research”/ or exp “implementation of research”/ or exp “organization of research”/ or  exp research institutes/ or exp research policy/ or exp research projects/ or exp research support/ or exp research teams/ or exp research workers/ or exp research grants/ or exp program evaluation/ or  exp health care costs/ or “cost benefit analysis”.sh. or Exp health policy/ |
| #11  (Combine) | 9 AND 10 |

 Table S4. Search Strategy for Web of Science

| Term Domain | Search Terms |
| --- | --- |
| #1  Assessing Impacts | AB= ((assess* or eval* or analy* or frame* or compar* or review* or explor* or identif*) NEAR/3 (impact or contribut* or payback or role or util* or outcom* or influenc* or output* or return* or effect* or productiv*) and (research* or grant* or portfolio* or invest* or fund*)) |
| #2  Frameworks | AB= (SIAMPI or “social impact assessment” or “productive interactions” or ASIRPA or PIPA or “participatory impact pathways analy*” or “Payback framework” or “research impact framework” or “research excellence framework” or “research assessment exercise” or “Canadian academy of health sciences” or “research quality framework” or “excellence in research Australia” or “measurement of research impact and assessment” or “Weiss logic model” or “program assessment rating tool” or “Canadian Institutes of Health Research framework” or “HTA Quebec model” or “research utilization model”) |
| #3  Science of science | TS = (“science of science” or “research on research”) |
| #4  Research utility | TS=((research NEAR/3 (util*)) or (research NEAR/3 (use or using or usag*)) or (evidence NEAR/3 (pract*))) |
| #5  Translating knowledge | TS=((translat* NEAR/3 (research)) or (translat* NEAR/3 (knowledge)) or “knowledge production”) |
| #6  Citation mapping | TS = (“citation mapping” or “bibliometric*) |
| #7  Combine | 1 OR 2 OR 3 OR 4 OR 5 OR 6 |
| #8  Grant funding | TS= (grant* or scheme* or scholarship* or portfolio* or studentship* or award* or fellowship* or research* or fund*) |
| #9  Combine | 7 AND 8 |
| #10  WOS Categories | WC = ((Medicine, Research & Experimental) OR  (Biotechnology & Applied Microbiology) OR (Health Care Sciences & Services) OR (Health Policy & Services) OR  (Health Policy & Services) OR (Social Sciences, Biomedical) OR  (Social Sciences, Interdisciplinary)) |
| #11  Combine | #9 AND #10 |

###

Table S5. Search Strategy for SCOPUS

| Term Domain | Search Terms |
| --- | --- |
| #1  Assessing Impacts | TITLE-ABS (((assess* OR eval* OR analy* OR frame* OR compar* OR review* OR explor* OR identif*) near/3 (impact OR contribut* OR payback OR role OR util* OR outcom* OR influenc* OR output* OR return* OR effect* OR productiv*) AND (research* OR grant* OR portfol* OR invest* OR fund* ))) |
| #2  Frameworks | TITLE-ABS ( ( siampi  OR “social impact assessment”  OR  “productive interactions” OR  asirpa OR  pipa  OR  “participatory impact pathways analy*” OR “Payback framework”  OR  “research impact framework”  OR  “research excellence framework”  OR  “research assessment exercise”  OR  “Canadian academy of health sciences”  OR  “research quality framework”  OR “excellence in research Australia”  OR “measurement of research impact and assessment”  OR  “Weiss logic model”  OR  “program assessment rating tool”  OR  “Canadian Institutes of Health Research framework”  OR  “HTA Quebec model”  OR  “research utilization model”) ) |
| #3  Science of science | TITLE-ABS (“science of science” or “research on research”) |
| #4  Research utility | TITLE-ABS ((research w/3 (util*)) or (research w/3 (us*)) or (evidence w/3 (pract*))) |
| #5  Translating knowledge | TITLE-ABS ((translat* w/3 (research)) or (translat* w/3 (knowledge)) or “knowledge production”) |
| #6  Citation mapping | TITLE-ABS (“citation mapping” or bibliometric*) |
| #7  Combine | 1 OR 2 OR 3 OR 4 OR 5 or 6 |
| #8  Grant funding | TITLE-ABS ((grant* or scheme* or scholarship* or portfolio* or studentship* or award* or fellowship* or research* or fund*)) |
| #9  Combine | 7 AND 8 |
| #10  “Categories” | TITLE-ABS ((“medical research” OR “health research” OR “biomedical w/3 research” OR ((“health care” OR “health policy” OR “social sciences”) W/3 research))) |
| #11  Combine | #9 AND #10 |
| #12  Limits by year | Limit to Pub Year 2000-2021 and (limit-to (“English”) |

Table S6. Search Strategy for Google Scholar

| Query # | Search Terms |
| --- | --- |
| 1 | ((“impact” or “contribution” or “payback” or “assessment” or “evaluation”) and (“health” or “society” or “research”) and (“research funding”))  2005-2021  *Note: Only the first 500 results from query 1 were searched.* |
| 2 | ((“impact” or “contribution” or “payback” or “assessment” or “evaluation”) and (“health” or “society” or “research”) and (“research funding”))  2005-2021, site:.gov |
| 3 | ((“impact” or “contribution” or “payback” or “assessment” or “evaluation”) and (“health” or “society” or “research”) and (“research funding”))  2005-2021, site:.ac.uk |
| 4 | ((“impact” or “contribution” or “payback” or “assessment” or “evaluation”) and (“health” or “society” or “research”) and (“research funding”))  2005-2021, site:.gov.au |
| 5 | ((“impact” or “contribution” or “payback” or “assessment” or “evaluation”) and (“health” or “society” or “research”) and (“research funding”))  2005-2021, site:.ca |

Table S7. Search Strategy for ProQuest Dissertations and Theses

| Query | Search Criteria |
| --- | --- |
| 1 | subject(“Public health”) AND ti((research* OR grant* OR portfol* OR invest* OR fund*))    Doctoral dissertations only, English  2000-2021  Public Health OR medicine OR health care management OR public policy OR epidemiology OR medical research OR public administration OR studies OR research OR economics OR health care OR health sciences OR funding OR oncology OR cancer |

Table S8. Search Strategy for LSHTM Theses

| Query | Search Criteria |
| --- | --- |
| 1 | Title matches any of “research, grant*, fund* , portfol*” AND item type matches any of “Thesis” |

Table S9. Search Strategy for NLM Bookshelf

| Query | Search Criteria |
| --- | --- |
| 1 | (research[title] AND (grant*[Title]) OR fund*[Title] or program*[title]))    Published in last 10 years |
